# Update on the juvenile systemic sclerosis inception cohort http://www.juvenile-scleroderma.com

**DOI:** 10.1186/1546-0096-12-S1-P49

**Published:** 2014-09-17

**Authors:** Ivan Foeldvari, Angela Wierk, Tadej Avcin, Juergen Brunner, Rolando Cimaz, Tillmann Kallnich, Maria Katsikas, Maria Teresa Terreri, Mikhail Kostic, Dana Nemcova, Flavio Sztajnbok, Kirsten Minden, Monika Moll

**Affiliations:** 1 Hamburger Zentrum fuer Kinder- und Jugendrheumatologie, Hamburger Zentrum fuer Kinder- und Jugendrheumatologie, Hamburg, Germany; 2 Hamburger Znenturm fuer Kinder- und Jugendrheumatologie, Hamburg, Germany; 3 Pediatric Rheumatology, Ljubjana, Slovenia; 4 Pediatric Rheumatology, Insbruck, Austria; 5 Pediatric Rheumatologiy, Florence, Italy; 6 Pediatric Rheumatology, Berlin, Germany; 7 Pediatric Rheumatology, Buenos Aires, Argentina; 8 Pediatric Rheumatology, Sao Paolo, Brazil; 9 Pediatric Rheumatology, St. Petersburg, Russian Federation; 10 Pediatric Rheumatology, Prague, Czech Republic; 11 Pediatric Rheumatology, Rio de Janeiro, Brazil; 12 Pediatric Rheumatology, Tuebingen, Germany

## Introduction

Juvenile systemic sclerosis (jSSc) is an orphan autoimmune disease. Currently only retrospective data is existing regarding the organ involvement and evolvement of the disease. Our project is the first project, where in a prospective manner and with a protocol regarding standardized assessment of the organ systems and quality of life in early jSSc patients are assessed.

## Objectives

to learn about the evolvement of juvenile systemic sclerosis

## Methods

Patients with less than 18 months of disease duration, after the first Non-Raynaud symptomatic, are prospectively assessed, using a standardized protocol.

## Results

We report the patient characteristics at time point 0, 6 and 12 months of their follow up. We present date on 25 patients. The mean follow up of the patients in the cohort are 3.5 years. No patient died during the follow up. Eighteen of the 25 patients were female. The mean age of the onset of Raynaud symptomatic was 10.4 years, the youngest patient was 2.0 years of age. The mean age at the onset of the non-Raynaud symptomatic were 11.0 years. 19 of the 25 have diffuse subtype, 6 of them have an overlap symptomatic, two of them associated with diffuse subtype. ANA positive were 20, and 8 of them were anti-Scl 70 positive. None of them was anticentromere positive The mean modified Rodnan Skin Score was at timepoint 0, 6 and 12 month 18.1, 15.1 (n=21) and 15.1. (n=17). Raynaud´s Phenomen occurred in 22/25 at time point 0 and 16 of 21 at time point 6 months and 12 of 17 at 12 months. 18 of 25 of them had

capillary changes already at time point 0. 7 of them had already ulcerations at time point zero, 9 of 21 at month 6 and 4 of 17 at months 12. 15 of them had cardiopulmonary involvement, at time point zero already, 9 of them had interstitial lung disease. 6 of 21 have cardiopulmonary involvement at month 6 and 7 of 17 at month 12 of follow up. Two of them have renal involvement at time point 0 and 3 at time point 6 and 12 months. 9 of 25 had gastrointestinal involvement, and 5 of them oesophageal involvement at time point zero, 3 from 21 at month 6 and 5 of 17 at 12 months. 22 of 25 have musculoskeletal involvement 19 of 21 at month 6 and 16 of 17 at 12 months.

## Conclusion

We present the data on the first 25 prospectively assessed patients with jSSc. The current recruitment data confirms that pediatric patients are different from the adult patients. There is a striking majority of diffuse patients with 76% and overlap feutures in 24% of the patients. None of the patients were anticentromere antibody positive. Unfortunately despite the prospective data collection, we miss some data. The collecition of patients and data is ongoing (http://www.juvenile-scleroderma.com).

## Disclosure of interest

None declared.

